# 胸腔镜肺叶切除术后肺容积减少与患者肺功能丢失的相关性分析

**DOI:** 10.3779/j.issn.1009-3419.2021.103.15

**Published:** 2022-01-20

**Authors:** 振州 翟, 军 赵, 畅 李, 成 丁, 春 徐

**Affiliations:** 1 215006 苏州，苏州大学附属第一医院胸外科 Department of Thoracic Surgery, First Affiliated Hospital of Suzhou University, Suzhou 215006, China; 2 200336 上海，上海交通大学医学院附属同仁医院急诊科 Department of Neurosurgery, Tongren Hospital, Shanghai Jiao Tong University School of Medicine, Shanghai 200336, China

**Keywords:** 胸腔镜肺叶切除术, 肺容积, 肺功能, Thoracoscopic lobectomy, Lung volume, Pulmonary function

## Abstract

**背景与目的:**

探讨不同部位的肺叶切除术后患者肺容积减少与患者肺功能损害程度的相关性。

**方法:**

本研究共纳入苏州大学附属第一医院2019年1月-2020年7月行胸腔镜肺叶切除术的131例患者（包括左肺上叶，左肺下叶，右肺上叶，右肺中叶，右肺下叶切除术；其中男性72例，女性59例）。为了比较患者术后肺功能与术前肺功能的差异，分别于术前7天和术后3个月、6个月及1年记录患者的肺功能测量值。采用1秒用力呼气量（forced expiratory volume in 1 second, FEV_1_）作为肺功能变化的主要评估参数。采用Mimics Research 19.0软件计算出患者的原始肺容积，各阶段存留的肺容积。分析患者肺容积在上述时间节点与患者肺功能变化的相关性。

**结果:**

术后患者FEV_1_较术前降低，下降程度与肺叶的切除体积呈正相关（其中左下肺下降较为明显）。值得注意的是，患者的肺功能降低程度在术后3个月、6个月与1年的差异无统计学意义。

**结论:**

肺叶切除术后肺组织的容积减少是患者肺功能减少的主要原因，以左肺下叶表现为著，最早可选定术后3个月作为肺叶切除患者残留肺功能的评估节点。

## Abstract (en)

**Background and objective:**

To investigate the correlation between the reduction of lung volume and the degree of lung function damage after lobectomy.

**Methods:**

A total of 131 patients (72 males and 59 females) who underwent thoracoscopic lobectomy in the First Affiliated Hospital of Suzhou University from January 2019 to July 2020 (including thoracoscopic resection of left upper lobe, left lower lobe, right upper lobe, right middle lobe and right lower lobe). In order to compare the difference between postoperative pulmonary function and preoperative pulmonary function, the pulmonary function measurements were recorded at 7 days before operation, and 3 months, 6 months and 1 year after operation. Forced expiratory volume in 1 second (FEV_1_) was used as the main evaluation parameter of pulmonary function. The original lung volume and the remaining lung volume at each stage were calculated by Mimics Research 19.0 software. The correlation between lung volume and lung function was analyzed.

**Results:**

FEV_1_ in postoperative patients was lower than that before operation, and the degree of decline was positively correlated with the resection volume of lung lobes (the maximum value was shown in the left lower lobe group). Significantly, there was no significant difference in the degree of pulmonary function reduction between 3 months, 6 months and 1 year after operation.

**Conclusion:**

The decrease of lung tissue volume after lobectomy is the main reason for the decrease of lung function, especially in the left lower lobe. And 3 months after lobectomy can be selected as the evaluation node of residual lung function.

如今，肺癌患者的数量增加引发外科手术切除的需求增大，术后患者的肺功能受损已成为影响临床医生决策的“绊脚石”。传统观点认为在治疗癌症的目标下存在一定的并发症是合理的，但时常发生的致命性、难治性的肺功能缺失逐渐成为临床医生面临的挑战^[1]^。肺切除术导致的肺功能丧失主要取决于手术切除的程度、切除的组织对于剩余组织的相对功能状态以及术前肺功能受损的程度^[2, 3]^。目前辅助影像技术的应用实现了术前对计划性肺实质切除术后的肺功能进行预测，并在术后根据预测进行了准确性监测。但是在保留最大数量的功能性肺组织的前提下进行肿瘤根治性切除的主流目标下，尚缺乏肺叶切除的肺体积与丢失的肺功能之间对应的相关性研究的证据。此外，不同部位肺叶切除后对患者肺功能的影响亦不明确^[4]^。本研究旨在探讨不同部位的肺叶切除术后患者肺容积减少与患者肺功能损害程度的相关性。

## 1 资料与方法

### 1.1 数据获取

纳入苏州大学附属第一医院2019年1月-2020年7月收治的行胸腔镜肺叶切除的患者，收集患者肺功能检查及胸部计算机断层扫描（Computed tomography, CT）数据。

### 1.2 选择标准

纳入标准：①年龄35岁-75岁；②胸腔镜下肺叶切除，相同肺叶相同手术方式；③分别于术前7日和术后3个月、6个月及1年完成患者肺功能随访。排除标准：①年龄 < 35岁或者 > 75岁；②术后出现严重的肺部感染；③合并严重心或者肾功能不全等其他重要脏器功能障碍；④胸腔镜中转开胸手术者。

### 1.3 分析方法

肺功能检查：分别于肺叶切除术前7日及术后3个月、6个月及1年对患者肺功能测试。当患者临床稳定时，进行肺功能检查，必要时使用支气管扩张剂治疗。所有患者均进行了完整的肺活量测量处理: 用力肺活量（forced vital capacity, FVC）、1秒用力呼气量（forced expiratory volume in 1 second, FEV_1_）、血流-容积曲线、总肺阻力（total pulmonary resistance R-tot）、通过支气管扩张试验反应、剩余肺容积（residual lung volume, RV）。同时在相应的时间对患者完成血气分析检查。影像学处理：采用东芝320排CT扫描，原始Dicom格式图像Mimics Research 19.0工作站，以1 mm的横断图像为基础采用软件进行不同阶段的肺容积计算（图 1）。

**图 1:**
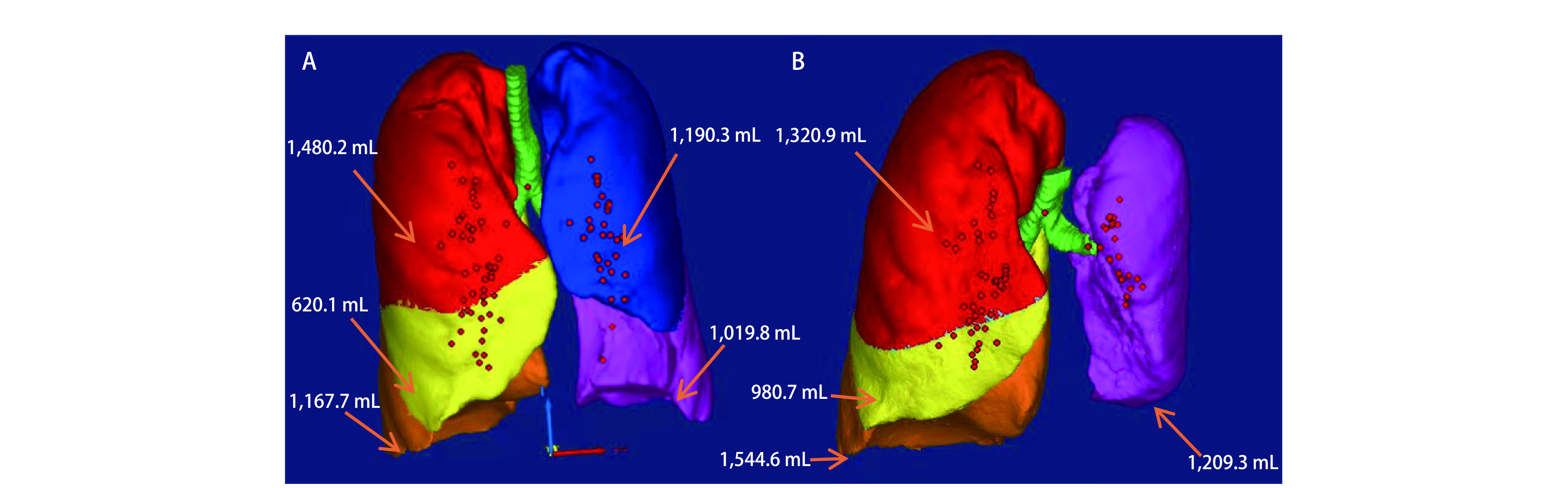
基于Mimics Research 19.0软件的术前术后患者不同肺叶容积的测量方法（以左上肺叶切除为例）A：术前；B：术后（注：红色：右肺上叶，黄色：右肺中叶，橙色：右肺下叶，蓝色：左肺上叶，粉色：左肺下叶）。 Different lung lobe volume labeling methods for preoperative and postoperative patients based on Mimics Research 19.0 software (left upper lobectomy as an example) A: preoperative, B: postoperative. (Note: red: right upper lobe, yellow: right middle lobe, orange: right lower lobe, blue: left upper lobe, pink: left lower lobe).

### 1.4 统计学分析

采用SPSS 22.0进行统计学分析，计量资料以均数±标准差（Mean±SD）表示，均进行正态性检验其是否符合正态分布。组间比较采用独立样本*t*检验。分类资料用例数（百分数）表示，组间比较采用卡方检验。采用*Pearson*相关性分析检验变量之间的关系。*P* < 0.05表示具有统计学差异。

## 2 结果

### 2.1 患者情况

本研究共纳入131例手术患者，其中男性72例，年龄（68.43±3.32）岁；女性59例，年龄（66.52±2.62）岁，手术切除类型及病例特征、患者性别、肿瘤大小和平均年龄分组情况如表 1所示。

**表 1:** 患者基本特征表（*n*=131） Basic characteristics of patients (*n*=131)

Category	Scope of resection	Number of patients (Male/Female)	Smoking history (Yes/No)	Tumor size (cm)	Age (yr)
1	Left upper lobe	21 (14/7)	17/4	2.8±1.9	68.17±3.21
2	Left lower lobe	38 (22/16)	29/9	3.5±2.3	66.12±4.24
3	Right upper lobe	20 (11/9)	13/7	2.3±1.6	70.58±3.01
4	Right middle lobe	17 (6/11)	12/5	3.0±1.4	65.73±2.54
5	Right lower lobe	35 (19/16)	23/12	3.2±1.8	69.25±3.73

### 2.2 肺功能情况

肺功能损害程度主要根据FVC（肺功能最常用的参数）（术前*vs*术后，*P* > 0.05）及FEV_1_（术前*vs*术后，*P* < 0.05）（肺功能最客观的参数，并指示通气性肺功能损害的类型和程度）来表示。患者术前术后血流-容积曲线及总肺阻力（R-tot）（通过支气管扩张试验反应）均不具有统计学差异（*P* > 0.05）。图 2显示了FVC平均值（包括正常值，术前、术后达到多少及其占正常值的百分比）；表 2反映了术后不同部位的肺功能下降情况与肺容积的相关性。

**图 2:**
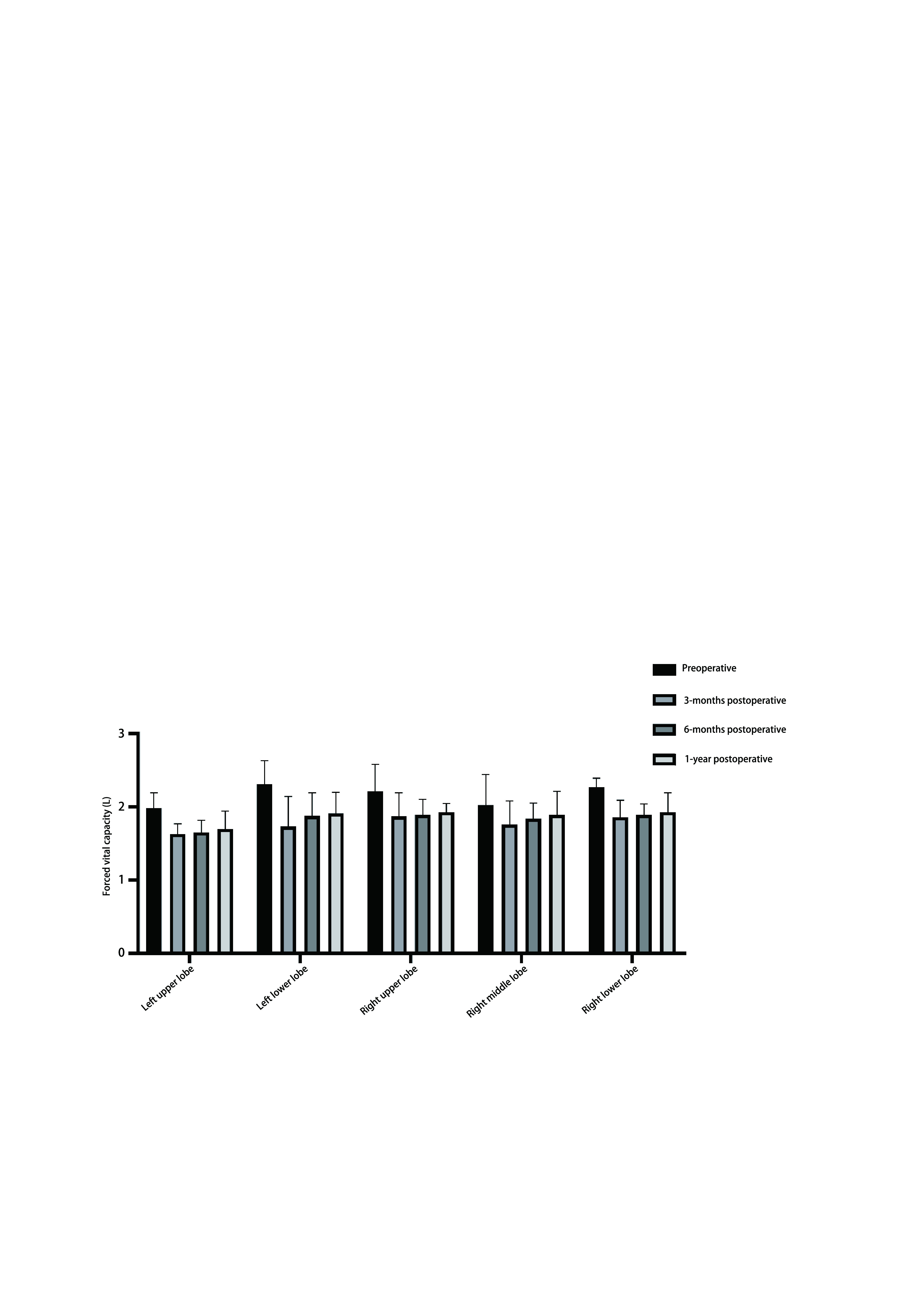
患者不同肺叶切除后FVC的实际情况 Actual situation of FVC after different lobectomy. FVC: forced vital capacity.

**表 2:** 患者肺叶不同切除部位FEV_1_与肺容积的相关性 Correlation between FEV_1_ and lung volume at different resection sites of lung lobes

Scope of resection	Preoperative FEV_1_ & volume (*R/P* value)	Postoperative FEV_1_ & volume (*R/P* value)		
		3 mon	6 mon	1 yr
Left upper lobe	0.442 (0.174)	0.363 (0.151)	0.381 (0.139)	0.341 (0.104)
Left lower lobe	0.521 (0.075)	0.642 (0.050)^*^	0.694 (0.048)^*^	0.712 (0.043)^*^
Right upper lobe	0.565 (0.071)	0.493 (0.068)	0.583 (0.064)	0.633 (0.058)
Right middle lobe	0.472 (0.192)	0.405 (0.132)	0.511 (0.109)	0.495 (0.115)
Right lower lobe	0.635 (0.064)	0.631 (0.066)	0.676 (0.059)	0.692 (0.048)^*^
FEV_1_: forced expiratory volume in 1 second; ^*^R: correlation coefficient; ^*^means statistically significant.				

## 3 讨论

尽管外科切除治疗是治疗肺部恶性肿瘤的最佳选择，但手术继发的患者肺功能受损是不能忽略的话题^[1, 4]^。由于缺乏呼吸储备，导致术后严重肺部并发症和死亡，这类患者的手术决策仍是临床医生面临的难题^[5]^。胸外科手术后的肺功能评估取决于类型、部位和程度引发了患者的肺功能受损。在评估实施胸部手术可施行性的过程中，不仅单单考虑到原发病灶的情况，更为重要的是综合性的心肺功能评估^[6]^。对于每个患者来说，术前评估必不可少，如果精心设计，肺叶切除术在70%-80%的病例中是可供选择的^[1, 6]^。

既往研究^[7]^发现，在进行肺切除时每减少30%-40%的肺体积将在术后3个月的时间内减少15%的肺功能。最近一项纳入103例患者进行肺叶切除的研究^[8]^表明，肺叶切除术后不能引起健康肺组织功能的代偿性增加。在我们的研究中，与术前相比，3个月后左肺上叶FEV_1_下降为0.35 L，比例为17.68%；左肺下叶FEV_1_下降为0.58 L，比例为25.11%；右肺上叶FEV_1_下降为0.34 L，比例为15.38%；右肺中叶FEV_1_下降为0.27 L，比例为13.3%；右肺下叶FEV_1_下降为0.41 L，比例为18.06%；这表明不同肺叶切除所致不同程度的肺功能下降对临床决策起至关重要的预警作用。亦有学者对68例肺切除患者进行术前、术后3个月和6个月肺功能测试发现，肺叶切除后患者的FVC、FEV_1_、肺总量（total lung capacity, TLC）、一氧化碳弥散量（diffusion capacity for carbon monoxide of the lung, DLCO）及最大耗氧量在3个月-6个月时仍明显低于术前，并且无论术前肺功能是否正常或受损，结果均一致^[9]^。这一研究证实，在术后6个月，肺功能的受损依然存在^[10]^。与上述研究发现一致，我们的结果显示在相应的时间（术后6个月及1年）患者的FEV_1_仍较正常值的水平低，这意味着术前及术后早期对患者进行可切除性评估将会成为肺叶切除术患者并发症降低及获益提高的关键^[11]^。有学者^[1]^认为在术前1个月内及在术后1周至3年（平均1.5年）进行肺功能检测发现，肺叶切除术患者的平均FEV_1_损失为150 mL，最大下降为870 mL。由此可见，高达70%的肺功能损害可能由于肺的病变部位切除所致，因此更需要谨慎的术前检查^[12]^。我们的研究发现，肺叶切除术后肺功能的最大下降发生在左肺下叶，其FEV_1_降低了0.58 L，与术前值（以正常值的百分比表示）相比，其下降幅度高达25.11%，这可能是由于该部位的肺组织体积较大所致。但是，我们的结果显示术前术后FVC、血流-容积曲线及总肺阻力（R-tot）（通过支气管扩张试验反应）均不具有统计学差异，这可能是因为目前医学诊断技术提高，符合肺叶切除的这一类患者自身肺功能基数条件较差，因而差异不明显。

实行肺叶切除患者的健侧肺组织较难实现代偿性增长^[13]^。其原因可能是：①成年人肺间质的不可再生性，理论上患者肺功能的缺失取决于患者肺组织切除的容积大小^[14]^；②在实施肺叶切除组，病人术前健康的肺组织已经长期发生了代偿性增长，可能达到了补偿限度，进而引起术后无进一步增长，因此残留量肺组织大小决定了肺叶切除患者的肺功能恢复情况^[13, 15]^；③肺叶切除术患者健侧肺组织过度增长使得同侧剩余肺组织的代偿增长受到限制^[15]^。在本研究中，我们发现残留体积越大的患者的FEV_1_下降越小，这种趋势呈现相关性，这一结论亦是对上述观点的论证。在今后的临床实践中，应该意识到这一现象，特别是边缘肺功能或是肺损毁的患者，术前筛查极为重要，以避免术后发生急性呼吸衰竭的并发症。传统观点认为肺叶切除术围术期的FEV_1_下降为术前的30%^[16]^，3个月后为17%，之后逐渐恢复^[14, 17]^。尽管认为在1年后患者肺功能可较3个月实现改善，但是我们的结果显示，患者3个月和6个月FEV_1_的差异与1年后不明显。造成这一结果差异的原因可能是目前主流观点认为节段切除术比肺叶切除术更能保留肺功能（不仅保留了肺叶，而且增加了同侧未手术肺叶的功能）的引导下，选择肺叶切除患者的病理分期靠后，患者肺基本情况差，进而所致代偿能力的降低。这一差异性更应引起临床医生的注意，因为此类患者术后早期肺不张，肺部感染等一系列肺部并发症风险较高，亦成为危害患者康复的最主要原因。此外，膈肌高度的变化的差异性分析，术中加强对膈肌和膈神经乃至淋巴结清扫过程中迷走神经的保护，术后加强腹式呼吸的锻炼，膈肌力量的加强，对患者3个月及1年肺功能的影响差异将会成为今后研究的新思路。

本研究尚存一定的不足：首先，切除术后患者的弥散功能同样也很重要；然后，术前术后肺容积变化有很多亚类可分析，如同侧肺、对侧肺容积变化情况差异，是否存在同侧和对侧肺分别代偿的改变。此外，肺功能自身也受很多因素影响，比如是否存在肿瘤性积液，是否存在慢性阻塞性肺疾病，是否有药物干预的影响，这些混杂因素研究应在今后的实验中进一步考虑。

根据我们的研究，肺叶切除术后肺组织的容积减少是患者肺功能减少的主要原因，以左下肺表现为著，最早于术后3个月可作为患者残留肺功能的评估节点，术前评估应充分考虑到这一点。因此，患者肺叶残留量的体积大小与患者肺功能缺失情况具有相关性这一重要问题亦应纳入术前考量的因素中。在术前肺功能评估中，应询问患者的心肺储备是否足以使患者在手术后存活，以及患者是否会因肺功能不足而长期无法日常生活。
